# A comparison of the effects of ursolic acid and l-leucine supplementation on IGF-1 receptor and AKT-mTOR signaling in response to resistance exercise in trained men

**DOI:** 10.1186/1550-2783-11-S1-P19

**Published:** 2014-12-01

**Authors:** David Church, Neil Schwarz, Mike Spillane, Sarah McKinley, Tom Andre, Darryn S Willoughby

**Affiliations:** 1 Baylor University, Waco, Texas, USA

## Background

Resistance exercise stimulates skeletal muscle protein synthesis (MPS) during post-exercise recovery due to up-regulation of the mammalian target of rapamycin (mTOR) signaling pathway. L-leucine supplementation is also known to stimulate MPS by activating mTOR signaling. However, recent research has discovered a natural compound called ursolic acid which also appears to stimulate MPS by activating the mTOR signaling pathway, and has been presumed to occur due to IGF-1 receptor (IGF-1R) up-regulation. Ursolic acid is a natural pentacyclic triterpenoid carboxylic acid that is widely found in apple skin and other fruits such as cranberries. The main purpose of this study was to compare the effects of a single dose of ursolic acid or L-leucine supplementation given immediately after resistance exercise on IGF-1 (a serum regulator of MPS) and the subsequent effects of IGF-1 on phosphorylating/activating its receptor (IGF-1RTyr1131). Furthermore, the purpose was to also determine the effects on signaling intermediates of MPS contained within the Akt/mTOR pathway (phosphorylated levels of AktThr308, mTORSer2448, p70S6KThr389).

## Methods

In a randomized, cross-over design, 9 apparently healthy, resistance-trained [regular, consistent resistance training (i.e. thrice weekly) for at least 1 year prior to the onset of the study], men between the ages of 18-30 volunteered to participate in this study and performed three separate testing sessions of lower-body resistance exercise involving 4 sets of 8-10 repetitions at 75-80% 1-RM on the angled leg press and knee extension exercises. Immediately after each resistance exercise session, participants orally ingested 3 grams (0.043 g/kg equivalent) of cellulose placebo (PLC), L-leucine (LEU), or ursolic acid (UA). A venous blood sample was obtained before, and 0.5, 2, and 6 hr post-exercise, whereas a vastus lateralis muscle biopsy was obtained before and 2 and 6 hr post-exercise. Each testing session was separated by 7 days to allow full recovery between sessions. Statistical analyses were performed utilizing separate two-way ANOVA for each criterion variable employing a probability level of ≤ 0.05. Consent to publish the results was obtained from all participants.

## Results

Using ELISA, no significant differences were observed among the three supplements for serum IGF-1 (p > 0.05). Also using ELISA, for skeletal muscle phosphoproteins, no significant differences existed among the three supplements for phosphorylated IGF-1R, Akt, and p70S6K (p > 0.05). However, the LEU supplement significantly increased phosphorylated mTOR compared to UA and PLC (p = 0.001).

## Conclusion

At the 3 g dose provided, ursolic acid was unable to increase IGF-1R signaling and, unlike L-leucine, ursolic acid had no positive effect on mTOR signaling activity. Therefore, ursolic acid appears to have no effect on mTOR activity when ingested immediately following resistance exercise.

